# Conformational Distribution of a Multidomain Protein Measured by Single-Pair Small-Angle X-ray Scattering

**DOI:** 10.1021/acs.jpclett.3c02600

**Published:** 2024-01-15

**Authors:** Honoka Kawamukai, Shumpei Takishita, Kazumi Shimizu, Daisuke Kohda, Koichiro Ishimori, Tomohide Saio

**Affiliations:** † Graduate School of Chemical Sciences and Engineering, Hokkaido University, Sapporo 060-8628, Japan; ‡ Graduate School of Medical Sciences, Tokushima University, Tokushima 770-8503, Japan; § Faculty of Education and Integrated Arts and Sciences, Waseda University, Tokyo 169-8050, Japan; ∥ Division of Structural Biology, Medical Institute of Bioregulation, Kyushu University, Fukuoka 812-8582, Japan; ⊥ Department of Chemistry, Faculty of Science, Hokkaido University, Sapporo 060-0810, Japan; # Institute of Advanced Medical Sciences, Tokushima University, Tokushima 770-8503, Japan; ∇ Fujii Memorial Institute of Medical Sciences, Institute of Advanced Medical Sciences, Tokushima University, Tokushima 770-8503, Japan

## Abstract

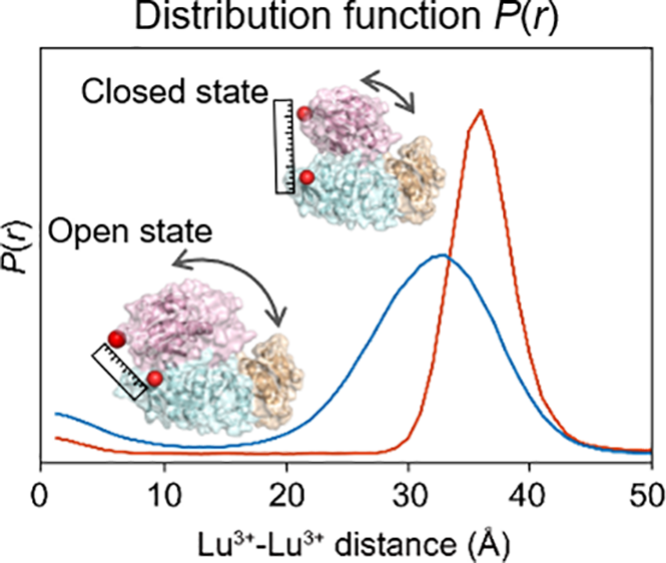

The difficulty in evaluating the conformational distribution of proteins in solution often hinders mechanistic insights. One possible strategy for visualizing conformational distribution is distance distribution measurement by single-pair small-angle X-ray scattering (SAXS), in which the scattering interference from only a specific pair of atoms in the target molecule is extracted. Despite this promising concept, with few applications in synthetic small molecules and DNA, technical difficulties have prevented its application in protein conformational studies. This study used a synthetic tag to fix the lanthanide ion at desired sites on the protein and used single-pair SAXS with contrast matching to evaluate the conformational distribution of the multidomain protein enzyme MurD. These data highlighted the broad conformational and ligand-driven distribution shifts of MurD in solution. This study proposes an important strategy in solution structural biology that targets dynamic proteins, including multidomain and intrinsically disordered proteins.

Conformational distribution is the key to understanding how proteins, especially multidomain proteins, function in solution. Multidomain proteins often regulate their activity by changing the population of multiple conformational states with varying domain orientations, contingent on ligand binding, post-translational modifications, and environmental changes.^1−4^ However, evaluating the conformational distribution of multidomain proteins in solution is not straightforward.^5,6^ Although recent technical developments in cryo-EM have expanded the application of the method to the structural determination of proteins with conformational variations, its application to those with continuous variations remains challenging. Additionally, the impact of freezing proteins for detection needs to be considered.^7^ Solution nuclear magnetic resonance (NMR) is useful for the structural study of proteins in solution, especially with the use of paramagnetic probes.^8,9^ On the other hand, NMR often suffers from resonance averaging because of the exchange among multiple conformational states, making data interpretation difficult. Small-angle X-ray scattering (SAXS) is one of the few methods for quantitatively assessing the conformational polydispersity of macromolecules, including multidomain proteins with flexible linkers and intrinsically disordered proteins (IDPs).^10^ However, the structural information obtained from typical SAXS analysis is of low resolution because each X-ray scattering between all of the atom pairs in the protein sums up to make a single SAXS curve; accordingly, the information is averaged. One way to obtain the conformational distribution of a protein is to exploit atoms with electron densities higher than those consisting of proteins. Scattering interference between heavy atoms on the protein can be extracted by contrast-matching^11^ or reference subtraction^12,13^ to determine the distance distribution between heavy atoms. If heavy atoms are fixed at specific sites on a protein, then the distance distribution of the heavy atoms reflects the conformational distribution of the protein. Previous studies using SAXS have demonstrated the measurement of the distance distribution for colloidal gold particles fixed at both ends of double-stranded DNA.^12,13^ A limitation was the size of the gold particle, 14 Å. The gold particle size is adequate for measuring the length of 10–35 bp DNA, with distance ranging from 50 to 140 Å, but is often too large to assess conformation changes in proteins. A demonstration using smaller particles was reported using two iodine atoms connected by a PEG chain, in which the distance distribution for a single pair of iodine atoms was obtained by reference subtraction.^14^ However, single-pair SAXS has not yet been used in protein conformational studies. Although SAXS distance measurements have been demonstrated for a calcium-binding protein substituted with four lead ions, the distance distribution was not obtained in this study presumably because of the complexity of the analysis owing to the four metals, resulting in six possible pair distances.^11^ Furthermore, its application is limited to metalloproteins; however, a new strategy applicable to non-metalloproteins is anticipated.

In this study, we evaluated the conformational distribution of a multidomain protein by single-pair SAXS using Lu^3+^ attached to two specific sites on the protein using a tag. Lu^3+^ has the highest number of electrons among the lanthanide ions, thus providing a larger X-ray scattering intensity compared to atoms consisting of proteins. Another advantage of lanthanide ions is that the techniques used to fix the ion to a specific site of the target protein have been extensively studied, especially in the field of biomolecular NMR.^8^ Among a number of lanthanide-binding tags, we used Caged Lanthanide NMR Probe 5 (CLaNP-5)^15,16^ attached to the protein through two arms to reduce the mobility of the tag with respect to the protein; accordingly, this provides more defined and reliable information about domain conformational states. X-ray scattering arising from Lu^3+^ was observed under contrast-matched conditions using 65% aqueous sucrose buffer, whose electron density matched that of the protein, resulting in the suppression of scattering from protein atoms at low angles.^11,17^

To demonstrate the conformational analysis of a multidomain protein using single-pair SAXS, we used a multidomain protein, MurD. MurD consists of three domains and is one of the ATP-driven Mur ligases responsible for peptidoglycan biosynthesis. Conformational states and ligand-driven conformational changes in MurD have been well-characterized in previous studies using X-ray crystallography, NMR, molecular dynamics (MD) simulations, and electron spin resonance (ESR).^18−23^ This makes MurD one of the best targets to demonstrate the application of single-pair SAXS. First, we evaluated the integrity of the strategy by a distance distribution measurement for the two lutetium ions (both fixed on the same domain of MurD) and confirmed that the result corresponded to the distance estimated from previous studies. Next, we conducted a conformational study of the full-length MurD with lutetium ions in domains 2 and 3. The data showed that MurD exists in multiple open conformations. On the other hand, inhibitor binding eliminates the conformations toward the closed conformation, highlighting the utility of single-pair SAXS for protein conformational studies in solution.

In this study, full-length MurD and MurD domains 1–2 (D12) were used to demonstrate single-pair SAXS. Prior to single-pair SAXS, MurD and D12 were subjected to size exclusion chromatography (SEC)-SAXS to test the oligomeric state and dispersity in solution. SAXS measurements followed by Guinier analysis on D12 and MurD resulted in an *R*_g_ of ∼21 Å and 23–24 Å, respectively (Figure 1A,B and Figure S1). The data are consistent with the predicted *R*_g_ values of 20.8 Å for D12 and 24.0 Å for MurD as obtained from the crystal structure (PDB ID:1e0d) of the MurD monomer using HullRad.^25^ The region comprising residues 1–302 was used to predict the *R*_g_ value of D12. The reasonable match between the experimental and predicted *R*_g_ values suggests that D12 and MurD exist as stable monomers under SAXS conditions. The monomeric states of D12 and MurD were also corroborated by size-exclusion chromatography with multiangle light scattering (SEC-MALS), which showed molar mass values consistent with D12 and MurD existing as monomers (Figure S2). Thus, the data showed that D12 and MurD exist as monomers in solution and are suitable for single-pair SAXS.

**Figure 1:**
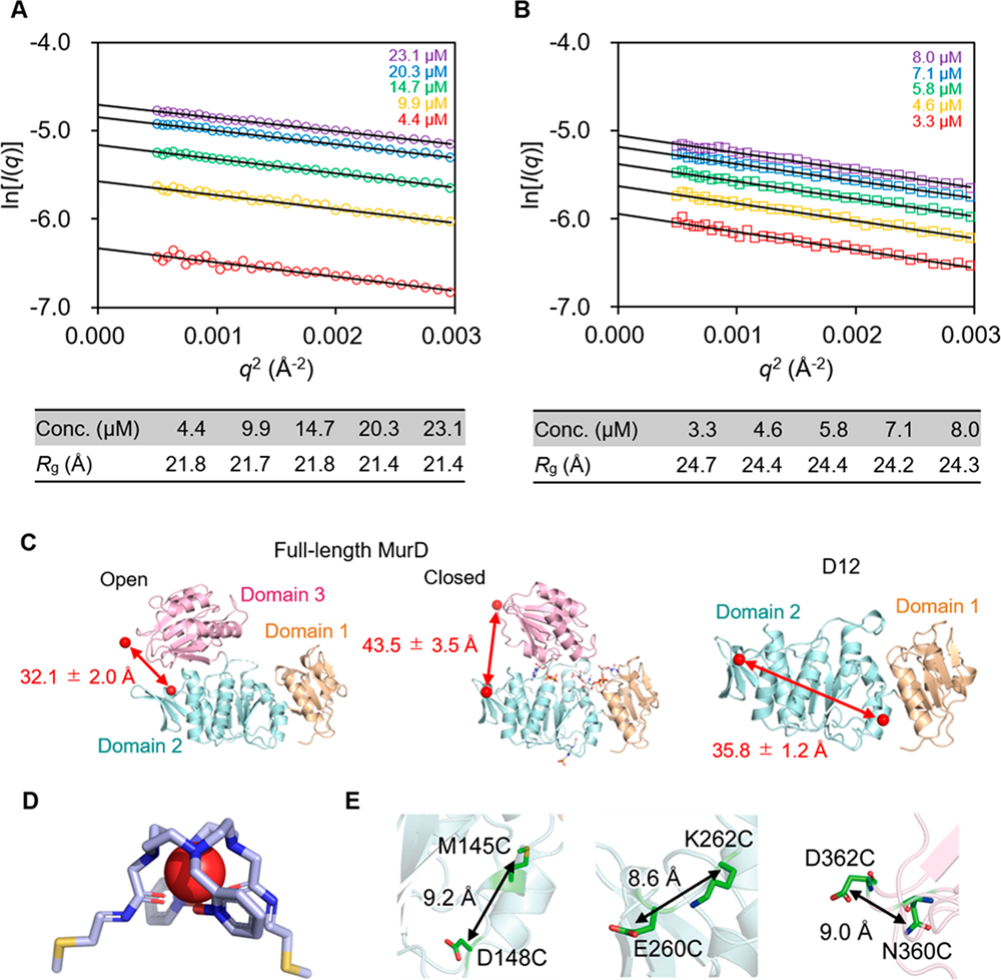
SAXS characterization and construct designs of MurD. (A, B) Guinier approximation, ln[*I*(*q*)] vs *q*^2^, of D12 and MurD scattering data in SEC-SAXS. The straight line gives the slope of data points from the least-squares method. (C) Lutetium tagging of MurD. The positions of the metals in MurD domains 1–2 M145C/D148C/C151A/E260C/K262C (D12_145–260_) and MurD E260C/K262C/N360C/D362C (MurD_260–360_) are shown as red spheres in the crystal structures of MurD (PDB: 1e0d, 3uag). Domains 1, 2, and 3 are colored yellow, blue, and pink, respectively. The distance between the lanthanoid ions, as estimated by crystal structures and pseudocontact shift (PCS) NMR analysis,^24^ is indicated by a red arrow. (D) Chemical structure of the Caged Lanthanide NMR Probe 5 (CLaNP-5) tag. (E) Close-up views of the positions of the lanthanoid ions fixed on MurD. The residues that were mutated to cysteine for ligation with the CLaNP-5 tag are indicated by red sticks.

To investigate the distance distributions of the multidomain protein MurD, we measured the distance between two lutetium ions fixed on domains 2 and 3 using SAXS (Figure 1C). Following the procedures described in previous reports,^9,16^ pairs of amino acid residues whose C_β_ atoms are located in a distance of 8–10 Å were selected and mutated to cysteine residues for attachment of CLaNP-5 via disulfide bonds (Figure 1D and E). Two MurD variants were constructed for the SAXS measurements: MurD domain 1–2 M145C/D148C/C151A/E260C/K262C (D12_145–260_) and full-length MurD E260C/K262C/N360C/D362C (MurD_260–360_) (Figure 1C–E). Because D12_145–260_ has two Lu^3+^ ions in domain 2, the distance distributions for D12_145–260_ are expected to reflect the local conformational variation of the tag and the Cys residues bridged to the tag. On the other hand, the distance distribution for MurD_260–360_, with Lu^3+^ ions in domains 2 and 3, was expected to reflect the conformational variations of domain 3 with respect to domain 2, in addition to local conformational variations (Figure 1C).

To test whether the Lu^3+^−Lu^3+^ distance information could be extracted, we performed SAXS measurement on D12_145–260_, in which CLaNP-5 tags containing Lu^3+^ were attached to the rigid regions of domain 2 (Figure 1C). To eliminate scattering from protein atoms, we next performed contrast-matched SAXS using a buffer containing 65% sucrose, which has the same electron density as the protein.^11^ Prior to contrast-matched SAXS, the effect of 65% sucrose buffer on the structures of D12 and MurD was evaluated by using circular dichroism (CD). Essentially identical CD spectra in the absence and presence of 65% sucrose were observed for D12 and MurD, indicating that the structures of D12 and MurD were preserved, even in 65% sucrose buffer (Figure S3).

Contrast-matched SAXS for the D12_145–260_ data showed an oscillating curve profile characteristic of scattering interference between the two atoms (Figure 2A). As a reference experiment, we also obtained the SAXS data for D12 without Lu^3+^ ions (Figure S4). Although the data show a slightly rugged profile presumably reflecting the nonuniform electron density distribution of the protein,^26,27^ the disturbance was much less significant compared to the oscillating curve profile for D12_145–260_ attached with Lu^3+^ ions. The data of the contrast-matched SAXS for D12_145–260_ attached with Lu^3+^ ions were fitted according to eq 1
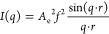
1where *I*(*q*) is the scattering intensity [*q* = 4π sin(θ)/λ, θ = one-half of the scattering angle; λ = X-ray wavelength], *A*_e_ is the scattering amplitude of an atom, *f* is the scattering factor, and *r* is the atomic distance. The data were fitted using eq 1 with the maximum entropy procedure coded in MATLAB, which was previously used for the analysis of gold-nanocrystal-labeled DNA fragments.^12,13^ The fitting using a distance range of 0–200 Å resulted in a major peak around 37 Å, with a couple of minor peaks above 50 Å (Figure 2B and Figure S5A).

**Figure 2:**
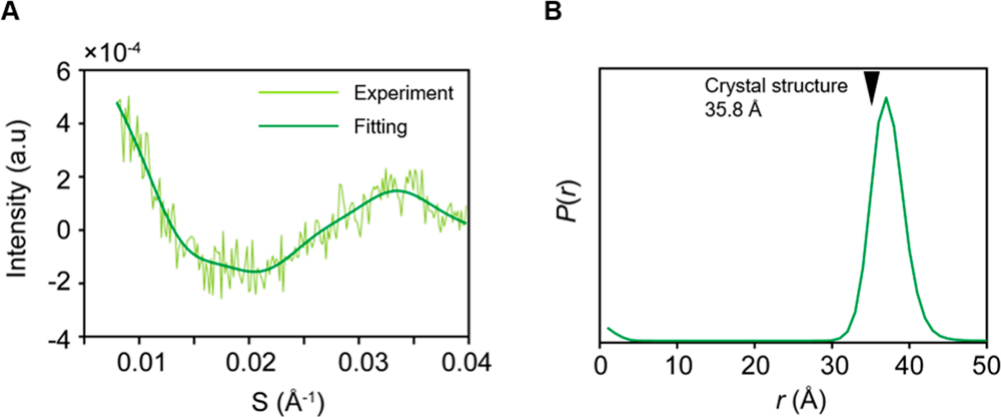
The distance between two points within D12_145–260_ was measured by using flow-SAXS. (A) SAXS scattering data and fitting. (B) Lu^3+^–Lu^3+^ distance distribution. The arrow indicates the distance between the lutetium ions expected from the crystal structures of MurD domain 1–2 (PDB: 3uag, 35.8 Å). The data were analyzed by using the MATLAB program.

The Lu^3+^–Lu^3+^ distance on D12_145–260_ [as estimated from crystal structure and pseudocontact shift (PCS) analysis]^9,24^ was 35.8 ± 1.2 Å; this agreed with the major peak of the SAXS-derived distance distribution curve. Because two Lu^3+^ ions are attached to the same domain, the width of the distribution should mainly be accounted for by the local conformational variation of the tag and the cysteine residues holding the tag. Additionally, the previous study exploiting ESR with double electron–electron resonance (DEER) measurement for D12_145–260_ showed the distance distribution having a peak top at 36.4 Å and full width at half-maximum (fwhm) of 7.7 Å;^24^ these are highly consistent with the SAXS-derived distance distribution, supporting the reliability of the distance measurement in this method. Note that the minor peaks that appeared in the 50–150 Å range can be explained by intermolecular contributions, given that the average distance between the centers of protein at the concentration of contrast-matched SAXS measurement (700 μM) is estimated as ∼130 Å. Thus, the data from contrast-matched SAXS on D12_145–260_ are highly consistent with the expected values and the results from other measurement techniques; therefore, we concluded that single-pair SAXS using contrast matching successfully extracted the distance distribution of Lu^3+^–Lu^3+^ from the protein.

The Lu^3+^–Lu^3+^ distance for MurD_260–360_ was measured to monitor the relative position of domains 2 and 3 of MurD in the absence and presence of the inhibitor *N*-({3-[({4-[(*Z*)-(2,4-dioxo-1,3-thiazolidin-5-ylidene)methyl]phenyl}amino)methyl]phenyl}carbonyl)-d-glutamic acid.^20^ Because the two lutetium ions were attached to domains 2 and 3, the Lu^3+^–Lu^3+^ distance distribution should reflect the position of domain 3 with respect to domain 2 (Figure 1C). The Lu^3+^–Lu^3+^ distance was expected to increase in the closed conformation (Figure 1C).

Contrast-matched SAXS of ligand-free MurD_260–360_ resulted in an oscillating SAXS curve, reflecting Lu^3+^–Lu^3+^-derived scattering interference (Figure 3A). As is the case of D12_145–260_, the data was fitted using eq 1 with the maximum entropy procedure coded in MATLAB. The fitting resulted in a broader distance distribution peak than that obtained for D12_145–260_ (Figures 2B and 3B, blue). The position of the peak top is close to the distance expected from the crystal structure of MurD in the open conformation (PDB:1e0d, 32.1 ± 2.0 Å)^9,22,24^ (Figures 1C and 3B, blue), suggesting that MurD exists in a variety of conformational states, including the open conformation seen in the crystal structure as a major state and other associated states.

**Figure 3:**
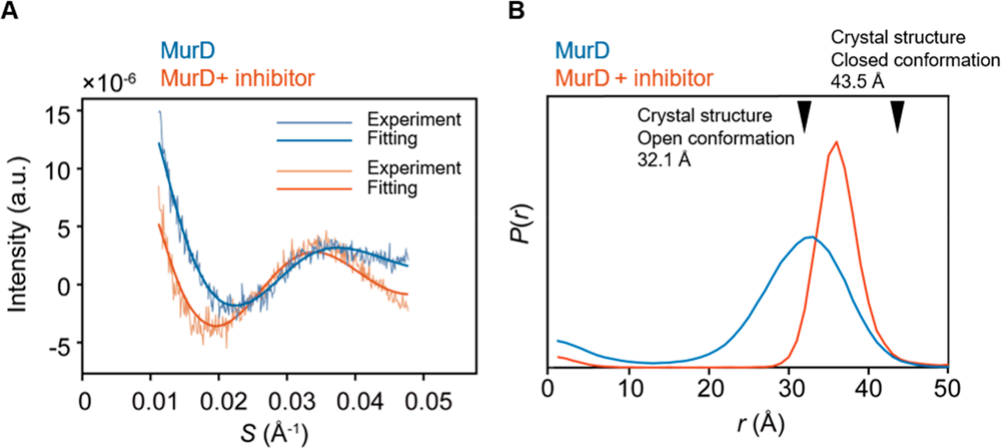
The distance between two points within MurD_260–360_ was measured by using flow-SAXS. (A) SAXS scattering data for MurD_260–360_ (blue) and inhibitor-bound MurD_260–360_ (red). (B) Lu^3+^–Lu^3+^ distance distributions. The insets show a wider distance distribution range of ≤50 Å. The arrows indicate the distance between the lutetium ions expected from the crystal structures of MurD in open (PDB: 1e0d, 32.1 Å) and closed (PDB: 3uag, 43.5 Å) conformations. The data were analyzed using the MATLAB program.

Contrast-matched SAXS of inhibitor-bound MurD_260–360_ also exhibited an oscillating SAXS curve. Nonetheless, the profile (especially the oscillation frequency and amplitude) was different from that of the ligand-free MurD (Figure 3A). The fitting resulted in a distance distribution with a major peak at a longer distance and a narrower width compared to that of ligand-free MurD_260–360_ (Figure 3B, Figure S5B and C). The narrower width of the peak for inhibitor-bound MurD_260–360_ indicated that inhibitor-bound MurD exists in more defined conformational spaces. The peak position at longer distances in the complex with the inhibitor suggests that the conformation of MurD changed from open to closed.

To verify the fitting analysis, theoretical SAXS curves were calculated from the normal distribution, as represented by eq 2
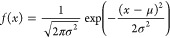
2where μ is the mean of expectation and is equal to the peak top of the distance distribution and σ is the standard deviation with the relationship fwhm = 2.35σ. The theoretical SAXS traces corresponding to the distance distributions for MurD_260–360_ in the absence and presence of the inhibitor (σ = 5.10 Å, μ = 33 Å and σ = 2.55 Å, μ = 36 Å) replicated the experimental data well (Figure 3 and Figure S6). Next, the reliable range of the distance distribution, apparent distance resolution to distinguish structures, was examined by comparing the experimental and calculated SAXS curves. The experimental SAXS data for D12_145–260_ were well reproduced by the calculated curve from the normal distribution of the distance corresponding to Figure 2B: μ = 37 Å and σ = 2.12 Å (Figure S7A and B). The calculated curves with varying μ showed that those with μ in a range of 37 ± 3 Å fit within the noise level of the experimental data, whereas those with μ = 37 ± 4 Å protrude from the experimental curve, suggesting that the reliable range of the peak top can be assumed as 37 ± 3 Å. The same evaluation was performed for the data of MurD_260–360_ in the absence and presence of the inhibitor (Figure S7C–F), resulting in the estimated reliable range of the peak top for MurD_260–360_ in the absence and presence of the inhibitor being 33 ± 2 and 36 ± 2 Å, respectively. With the above evaluations, we concluded that Lu^3+^–Lu^3+^ single-pair SAXS allowed us to measure the distance distribution of MurD and the inhibitor-induced changes in the distance distribution. Collectively, distance distribution measurements by single-pair SAXS revealed the conformational distribution and ligand-driven conformational changes of the multidomain protein MurD in solution, thus highlighting the usefulness of this method for evaluating the conformational distribution of proteins in solution.

Despite the importance of the conformational distribution of proteins for their function, their evaluation in solution is often not straightforward. This study demonstrates that single-pair SAXS using lanthanide ions as a scattering source can be used to evaluate the distance distribution of a multidomain protein in solution. Scattering interference between lanthanide ions was selectively observed by suppressing those from protein atoms using contrast-matched SAXS in 65% sucrose buffer. The distance distribution obtained for D12_145–260_ with the two lanthanide ions in domain 2 showed a major peak with peak top at 37 Å with an estimated reliable range of ±3 Å and was consistent with the distance expected from the crystal structure if the local conformational variation around the tag was considered, indicating the ability of this method to measure the distance between two specific points on the protein (Figure 2). Distance measurement for MurD_260–360_ in the ligand-free state showed a broader distance distribution than that observed for D12_145–260_, showing a variety of conformational states of MurD. This is consistent with the observations of previous studies using PCS-NMR, ESR, and MD simulation^9,24,28^ (Figure S8). The position of the peak top, 33 Å with an estimated reliable range of ±2 Å, is also consistent with the crystal structure in the open conformation and previous data from ESR and MD simulations (Figure 1C and Figure S8A). MurD_260–360_ in complex with the inhibitor showed a narrower distance distribution at longer distances compared to that of ligand free MurD_260–360_, indicating that the inhibitor induces a conformational change toward more closed and defined conformations. It should be noted that the position of the peak top for inhibitor-bound MurD_260–360_ was located at 36 Å with an estimated reliable range of ±2 Å (Figure S7E and F) that is distinct from the crystal structure of MurD in the closed conformation^19^ (Figure 1C) and the previous ESR distance measurement for frozen protein at 10 K (Figure S8B).^24^ These observations suggest that the most populated state of inhibitor-bound MurD_260–360_ in solution is different from that seen in the ESR measurement or in the crystal structure. Although the conformational difference of MurD between the crystal and solution has been suggested by other methods (including ESR-DEER measurement^24^ and MD simulation^28^), the current data from single-pair SAXS provide the first experimental views of the MurD conformation in aqueous solution. However, a current limitation is the difficulty in visualizing the actual conformational state of inhibitor-bound MurD_260–360_ in solution, mostly because of the limited structural information from distance measurement for a single pair. Further study including the multiple sets of distance measurement for lanthanide ions attached on the other positions would be anticipated.

Using a lanthanide-binding tag, this study successfully demonstrated the application of contrast-matched single-pair SAXS as a tool for visualizing the conformational distribution of a specific protein site. One of the advantages of this strategy is that the distance distribution can be measured for any desired point on the protein as long as the lanthanide can be fixed by the tag. Although we demonstrated the application of the CLaNP-5 tag, the same strategy can be exploited with other lanthanide-binding tags, including single-arm tags for easier design of the fixing points and tags with nonreducing linkages with the protein for measurement under reduced conditions.^8^ These facts highlight the generality and significance of this method for protein structural studies. For application of this method to other proteins, one may be concerned about the impact of 65% sucrose on the structure of the protein. It should be important to evaluate the structure of the target protein in 65% sucrose by other methods, including CD.

A possible future application of single-pair SAXS is in the structural study of intrinsically disordered proteins (IDPs). Owing to the importance of IDPs in many biological events, including liquid–liquid phase separation to form membraneless organelles, the need for information about their conformational distribution in solution is increasing. However, as with multidomain proteins, it can be difficult to adapt conventional structural analysis methods because of the structural flexibility of IDPs. By combining information about the distance distribution between two points in an aqueous solution with information obtained from NMR, spectroscopy, and MD simulations, more detailed structural information on IDPs can be obtained.

## Experimental Methods

### Preparation of CLaNP-5

CLaNP-5 was synthesized, purified, and chelated with Lu^3+^ as described in previous reports.^9,16,29^

### Protein Sample Preparation

*Escherichia coli* full-length MurD (1–437) and domains 1–2 (1–302) were cloned into pGBHPS,^30^ expressed in *E. coli* strain BL21 (DE3), and purified as described in a previous report.^9^ For SAXS measurements, MurD domains 1–2 M145C/D148C/C151A/E260C/K262C (D12_145–260_) and MurD E260C/K262C/N360C/D362C (MurD_260–360_) were prepared using a previously described procedure.^9^ Note that D12_145–260_ contains a mutation in C151A to avoid intramolecular disulfide bond formation with D148C. The CLaNP-5 tag was attached to the protein by mixing the protein and tag in a 1:2.2 ratio for 15 min on ice, followed by gel filtration. The data showed that the tags were attached to a specific position of MurD with high efficiency. Although MurD has seven cysteine residues (e.g., C20, C99, C151, C208, C227, C368, and C413), all cysteine residues have a thiol group buried in the protein and do not react with the CLaNP-5 tag.^9^ To replace the protein buffer with 65% (w/v) sucrose buffer, MurD and 65% sucrose buffer were mixed in a 1:1 volume and dialyzed overnight.^11^ For more accurate contrast match, 30 μL of protein solution and 1.5 mL of 65% sucrose buffer were added to a sitting drop plate (well capacity, 1.5 mL; post capacity, 40 μL; plate dimension, approximately 15.0 cm × 10.8 cm × 2.2 cm; HAMPTON RESEACH., Ltd., Osaka, Japan) and incubated overnight.

### Flow-SAXS Measurement

Contrast-matched SAXS for D12_145–260_ was recorded at beamline BL-10C of the Photon Factory (PF; Tsukuba, Japan). The X-ray wavelength was 1.1 Å, and the sample–detector distance was 1.0 m. Data were collected using a PILATUS 300 K detector at 20 30 s exposures per flow. The data from six flows were collected and averaged. A silver behenate standard was used to locate the beam center and calibrate the scattering angle values. Data reduction was performed using the SAngler software.^31^ The measurements were performed in a 1.25 mm path length flow cell with fused quartz windows (2.5 mm × 6 mm × 0.02 mm, Unisoku Co., Ltd., Osaka, Japan). Silicone rubber was used to hold the windows in place. The flow rate was set to 0.33 μL min^–1^ to reduce irradiation damage. The cell temperature was set to 20 °C. Buffer scattering was recorded as a reference. After the sample measurements, the cells were washed with a detergent solution and water.

As a reference, SAXS for D12 without lanthanide ion in 65% sucrose solution was recorded at the beamline BL-10C of the Photon Factory (PF; Tsukuba, Japan). The X-ray wavelength was 1.1 Å, and the sample–detector distance was 1.0 m. Data were collected using a PILATUS3 2M detector at 20 30-s exposures per flow. The data from 12 flows were collected and averaged. A silver behenate standard was used to locate the beam center and calibrate the scattering angle values. Data reduction was performed using the SAngler software.^31^ The measurements were performed in a 1.25 mm path length flow cell with fused quartz windows (2.5 × 6 × 0.02 mm^3^, Unisoku Co., Ltd., Osaka, Japan). Silicone rubber was used to hold the windows in place. The flow rate was set to 0.33 μL min^–1^ to reduce irradiation damage. The cell temperature was set to 20 °C. Buffer scattering was recorded before and after each sample. After the sample measurements, the cells were washed with a detergent solution and water.

Contrast-matched SAXS for MurD_260–360_ was recorded at beamline BL-6A of the Photon Factory (PF; Tsukuba, Japan). The X-ray wavelength was 1.5 Å, and the sample–detector distance was 1.0 m. Data were collected using a PILATUS3 1M detector with 20 20-s exposures per flow. The data from the five flows were collected and averaged. A silver behenate standard was used to locate the beam center and calibrate the scattering angle values. Data reduction was performed using the SAngler software.^31^ The measurements were performed in a 1.25 mm path length flow cell with fused quartz windows (2.5 mm × 6 mm × 0.02 mm, Unisoku Co., Ltd., Osaka, Japan). Silicone rubber was used to hold the windows in place. The flow rate was set to 6 μL min^–1^ and measured at 0 °C to reduce irradiation damage. Buffer scattering was recorded before and after each sample. After the sample measurements, the cells were washed with a detergent solution and water.

### SEC-SAXS Measurement

SEC-SAXS for MurD and D12 was recorded at beamline BL-10C of the Photon Factory (PF; Tsukuba, Japan). The X-ray wavelength was 1.1 Å, and the sample–detector distance was 1.0 m. Data were collected using a PILATUS 2M detector at 20 30-s exposures per sample. A silver behenate standard was used to locate the beam center and calibrate the scattering angle values. Data reduction was performed using the SAngler software.^31^ The measurements were performed in a 1.25 mm path length flow cell with fused quartz windows (2.5 × 6 × 0.02 mm^3^, Unisoku Co., Ltd., Osaka, Japan). Silicone rubber was used to hold the windows in place. The flow rate was set at 0.05 mL min^–1^. The cell temperature was set to 20 °C.

### Distance Distribution Analysis

To extract distance distribution information from the SAXS data, the data from contrast-matched SAXS were fitted with eq 1 using a modified version of the MATLAB program used in a previous study.^11^ The original program was used to study gold-nanocrystal-labeled DNA fragments.^12,13^ In the fitting of the MurD data, a linear baseline correction was added. To consider the reliable range of the distance information, the SAXS curves were regenerated from the *P*(*r*) function. The *P*(*r*) function was assumed to be a normal distribution. The peak top corresponded to mean (μ), and standard deviation (σ) was obtained from fwhm = 2.35σ. The SAXS curve was estimated for varying μ, with the range up to ±5 Å to search for the fitted region.

### SEC-MALS Measurement

SEC-MALS was performed using a DAWN HELEOS8+ (Wyatt Technology Corporation, Santa Barbara, CA, USA), high-performance liquid chromatography pump LC-20AD (Shimadzu, Kyoto, Japan), refractive index detector RID-20A (Shimadzu), and UV–vis detector SPD-20A (Shimadzu), located downstream of the Shimadzu liquid chromatography system connected to a PROTEIN KW-803 gel filtration column (Cat. No. F6989103; Shodex, Tokyo, Japan). Differential RI (Shimadzu) downstream of MALS was used to determine the protein concentrations. The running buffer used contained 20 mM Tris–HCl (pH 7.2) and 100 mM NaCl for D12 and 20 mM Tris–HCl (pH 7.2) and 200 mM NaCl for MurD. Approximately 100 μL of the sample was injected at a flow rate of 1.0 mL min^–1^. Data were analyzed by using ASTRA version 7.0.1 (Wyatt Technology Corporation). Molar mass analysis was also performed over half of the width of the UV peak top height.

### Circular Dichroism Spectrometer

The CD spectra were recorded using a JASCO J-1500 CD spectrometer (Tokyo, Japan) with 0.1 mm path length cuvettes at 25 °C in 20 mM Tris–HCl (pH 7.2), 200 mM NaCl, and 65% (w/v) sucrose buffer. Each spectrum represents an integration of four consecutive scans from 190 to 260 at 1.0 nm intervals, with a scan speed of 20 nm/min. The concentrations of MurD and MurD in 65% sucrose buffer were 34 and 27 μM, respectively.

## Supporting Information Available

Data for SEC-SAXS and SEC-MALS on D12 and MurD, circular dichroism on D12 and MurD in the absence and presence of 65% sucrose, contrast-matched SAXS on D12_145–260_ attached with the Lu^3+^ tags and D12, Lu^3+^–Lu^3+^ distance distributions for D12_145–260_ and MurD_260–360_, superimposition of theoretical SAXS curves, evaluation of the reliability of distance information from contrast-matched SAXS curves, and comparison of the SAXS-derived distance distributions with those estimated from ESR and MD simulation (PDF)
