# Supplementary material for: Conformational Distribution of a Multidomain Protein Measured by Single-Pair Small-Angle X-ray Scattering
Source: J Phys Chem Lett. 2024 Jan 15;15(3):744–50. doi: 10.1021/acs.jpclett.3c02600 (PMC10823528; doi:10.1021/acs.jpclett.3c02600)
Supplement: Supplementary file 1 — jz3c02600_si_001.pdf [file jz3c02600_si_001.pdf]

Supporting Information for Conformational Distribution of a Multidomain Protein Measured by Single-Pair Small-Angle X-Ray Scattering

Honoka Kawamukai[1,2], Shumpei Takishita[1], Kazumi Shimizu[3], Daisuke Kohda[4],

Koichiro Ishimori[1,5], and Tomohide Saio*[2,6,7]*

1. Graduate School of Chemical Sciences and Engineering, Hokkaido University, Sapporo, 060-8628, Japan

2. Graduate School of Medical Sciences, Tokushima University, Tokushima 770-8503, Japan

3. Faculty of Education and Integrated Arts and Sciences, Waseda University, Tokyo 169-8050, Japan

4. Division of Structural Biology, Medical Institute of Bioregulation, Kyushu University, Fukuoka 812-8582, Japan

5. Department of Chemistry, Faculty of Science, Hokkaido University, Sapporo, 060-0810, Japan

6. Institute of Advanced Medical Sciences, Tokushima University, Tokushima 770-8503, Japan

7. Fujii Memorial Institute of Medical Sciences, Institute of Advanced Medical Sciences, Tokushima University, Tokushima 770-8503, Japan

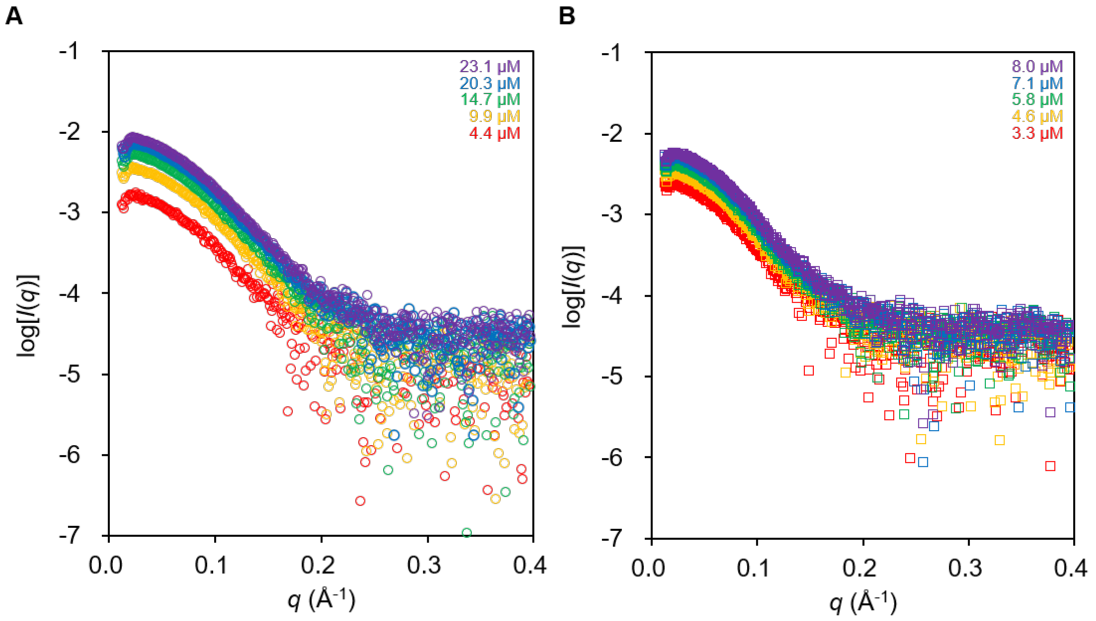

Figure S1. Monitoring monodisperse state at each concentration of D12 and MurD by using SEC-SAXS.

(A) SAXS curves for D12 in different concentration. The concentration of each is 4.4 µM (red), 9.9 µM (orange), 14.7 µM (green), 20.3 µM (blue) and 23.1 µM (purple).

(B) SAXS curves for MurD in different concentration. The concentration of each is 3.3 µM (red), 4.6 µM (orange), 5.8 µM (green), 7.1 µM (blue), 8.0 µM (purple).

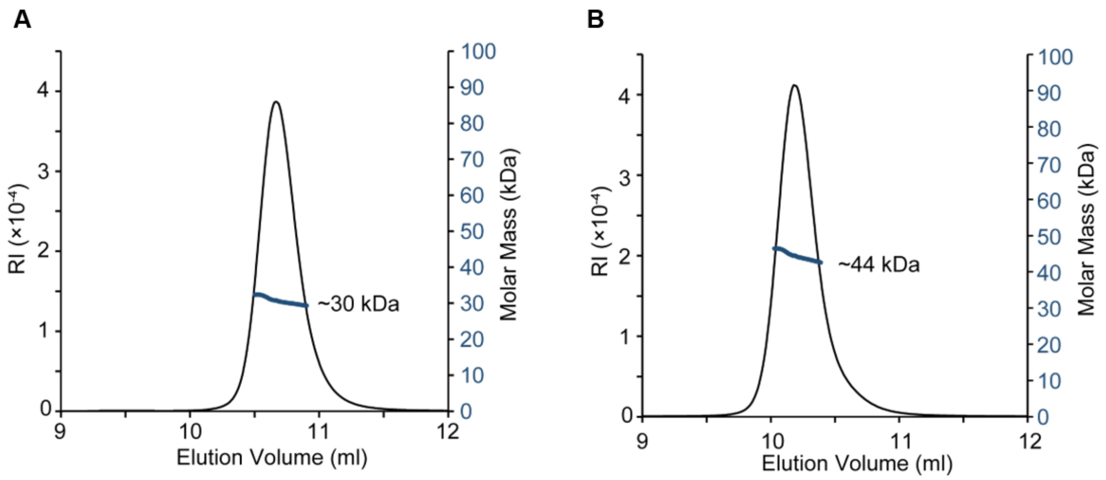

Figure S2. Monitoring monodisperse state of D12 and MurD by using SEC–MALS. The sample is D12 (A) and MurD (B).

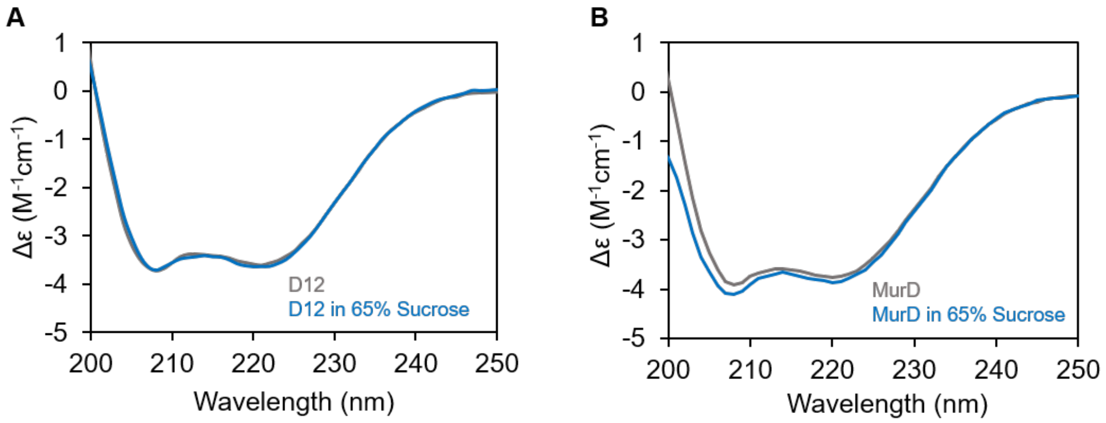

Figure S3. Monitoring 65% Sucrose-dependent secondary structural change of D12 (A) and MurD (B) by using Circular Dichroism spectrometer.

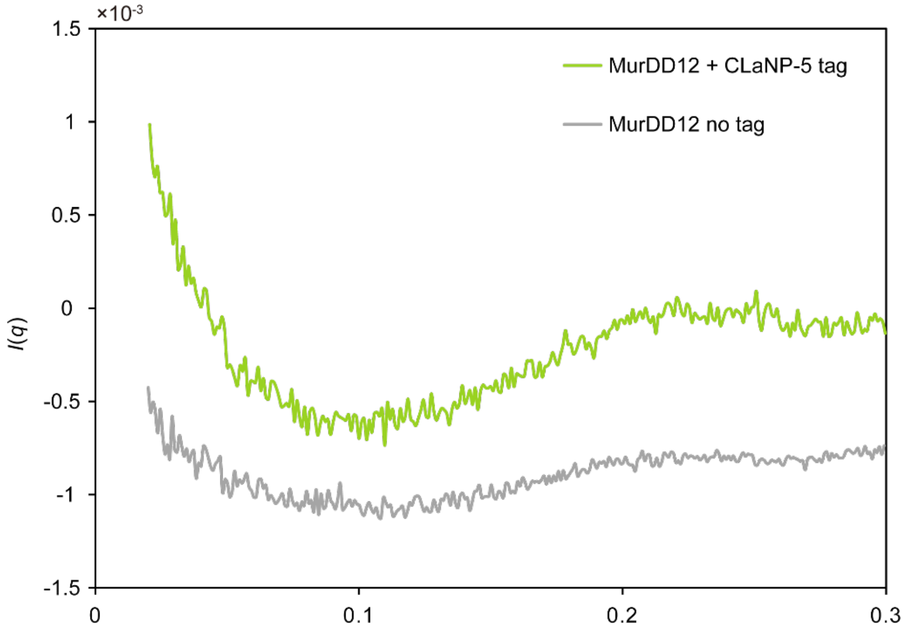

Figure S4. Absolute scattering data for $D12_{145\text{-}260}$ attached with the Lu^{3+} tags (light green) and D12 with no tag (gray).

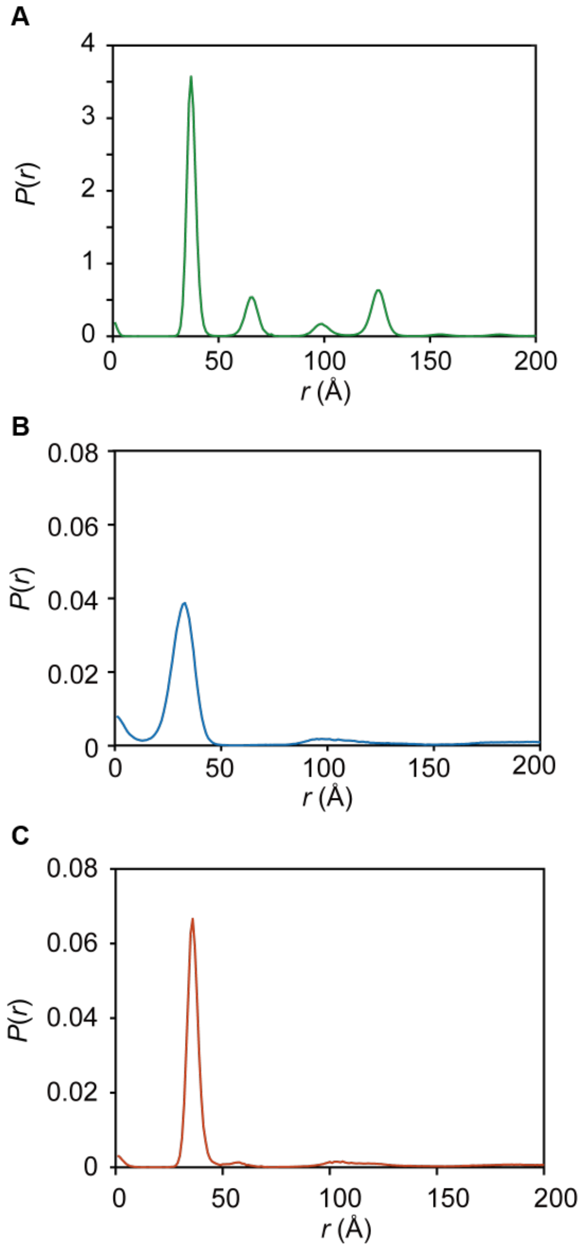

Figure S5. Lu^{3+}–Lu^{3+} distance distributions shown in 0–200 Å range. The data were analyzed using the MATLAB program. The sample is $D12_{145-260}$ (A), $MurD_{260-360}$ (B) and Inhibitor binding $MurD_{260-360}$ (C).

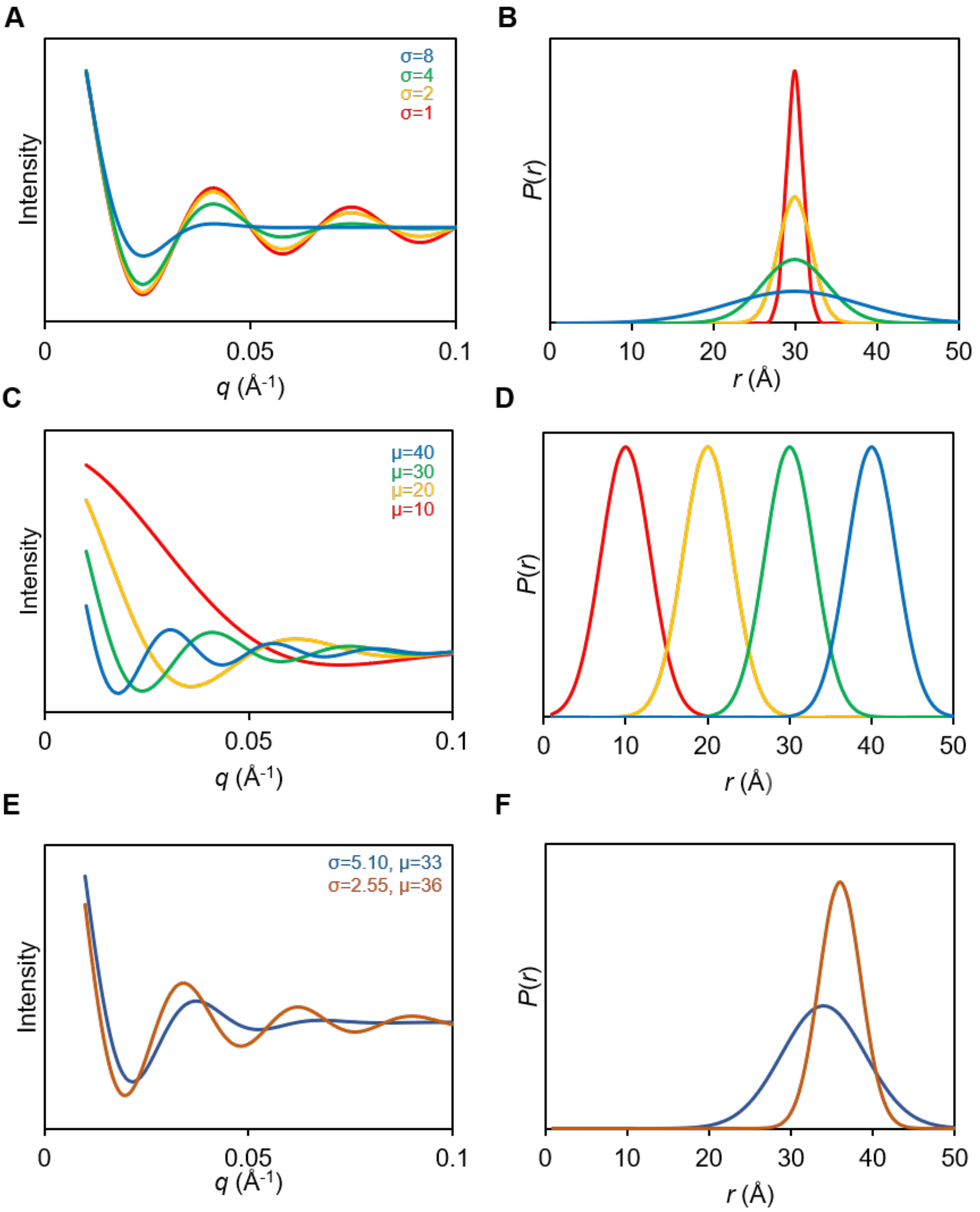

Figure S6. Superimposition of theoretical SAXS traces (A), (C), (E) and distance distributions (B), (D), (F).

(A, B) Variations in standard deviations of the Gaussian distribution: σ = 1 (red), 2 (orange), 4 (green), 8 (blue).

(C, D) Variations in means of the Gaussian distribution: μ = 10 (red), 20 (orange), 30 (green), 40 (blue).

(E, F) The theoretical SAXS traces corresponding to the distance distributions for $MurD_{260\text{-}360}$ in the absence (red) and presence of the inhibitor (blue) (σ = 5.10 Å, μ=33 Å and σ = 2.55 Å, μ=36 Å).

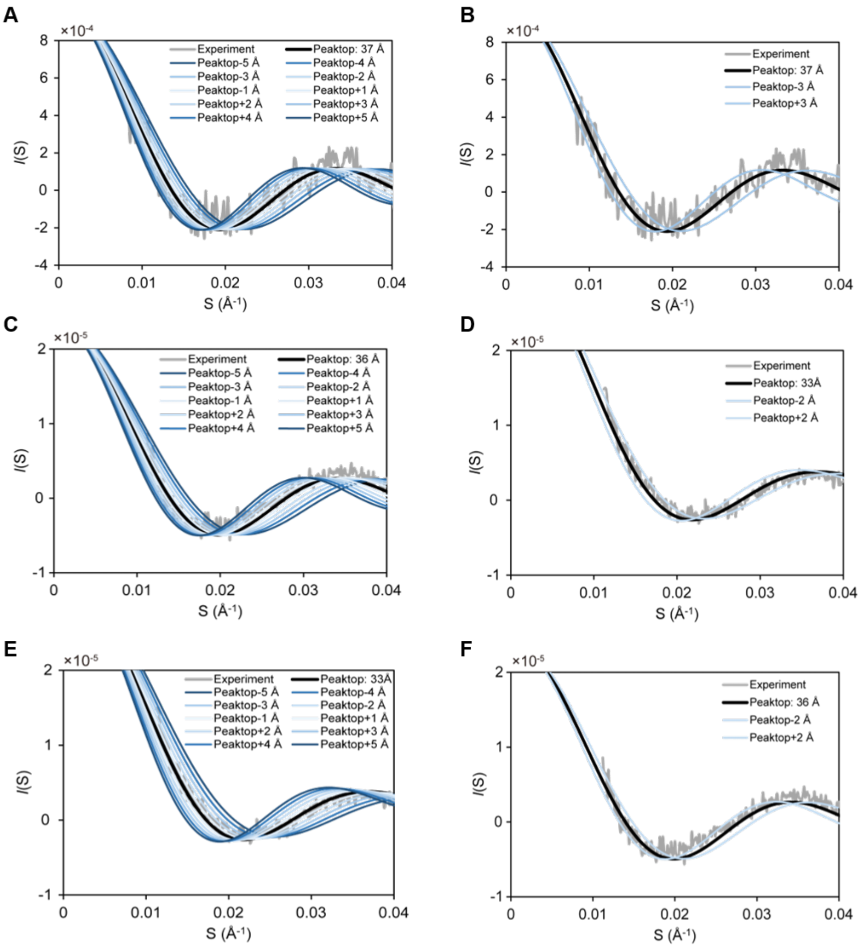

Figure S7. Evaluation of the reliable distance range for the distance information. The experimental data were compared with theoretical SAXS traces assuming Gaussian distribution with varying mean (μ) corresponding to the peak top of the distance distribution peak.

(A, B) The experimental SAXS data of $D12_{145\text{-}260}$ superimposed with theoretical SAXS trace calculated from Gaussian distribution with σ = 2.12 Å (FWHM = 5 Å) and μ = 37 Å that correspond to the major peak in the distance distribution (Figure 2B). The theoretical SAXS traces with μ shifted up to ± 5 Å (A) or μ shifted to ± 3 Å (B) are also shown. Considering the noise level, the calculated curves with μ shifted up to ± 3 Å can fit in the experimental data.

(C, D) The experimental SAXS data of apo $MurD_{260\text{-}360}$ superimposed with theoretical SAXS traces calculated from Gaussian distribution with σ = 5.10 Å (FWHM = 12 Å) and μ = 33 Å that correspond to the major peak in the distance distribution (Figure 3B). The theoretical SAXS traces with μ shifted up to ± 5 Å (C) or μ shifted to ± 2 Å (D) are also shown. Considering the noise level, the calculated curves with μ shifted up to ± 2 Å can fit in the experimental data.

(E, F) The experimental SAXS data of inhibitor binding $MurD_{260\text{-}360}$ superimposed with theoretical SAXS traces calculated from Gaussian distribution with σ = 2.55 Å (FWHM = 6 Å) and μ = 36 Å that correspond to the major peak in the distance distribution (Figure 2B). The theoretical SAXS traces with μ shifted up to ± 5 Å (E) or μ shifted to ± 2 Å (F) are also shown. Considering the noise level, the calculated curves with μ shifted up to ± 2 Å can fit in the experimental data.

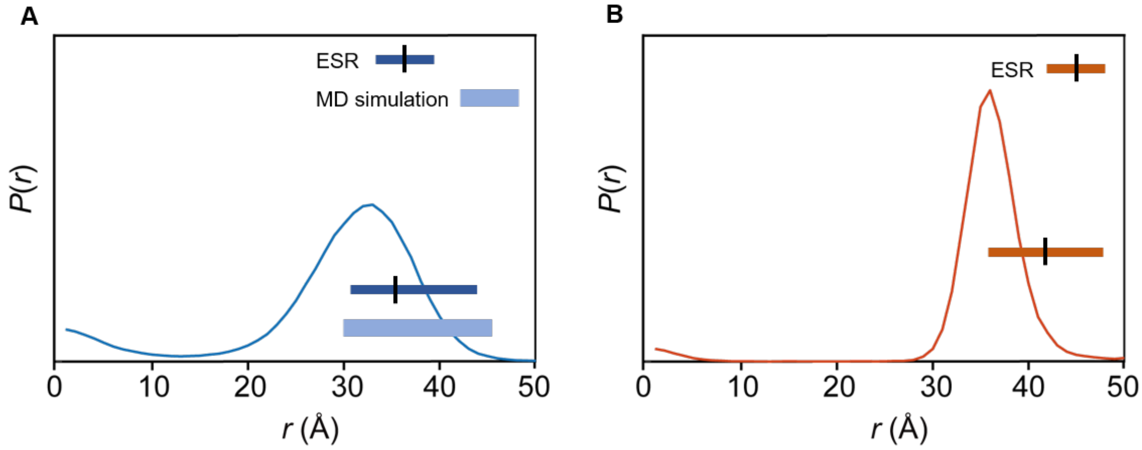

Figure S8. Comparison of the distance distribution of MurD$_{260\text{-}360}$ from SAXS, ESR, and MD simulation.

(A) SAXS-derived Lu^{3+}– Lu^{3+} distance distribution of apo MurD$_{260\text{-}360}$ (blue line), shown with the distance range derived from ESR distance measurement (blue bar with black line) and MD simulation (pale blue box).

(B) SAXS-derived Lu^{3+}– Lu^{3+} distance distribution of inhibitor-bound MurD$_{260\text{-}360}$ (dark orange line), shown with the distance range derived from ESR distance measurement (dark orange bar with black line). The black line in the bar representing ESR-derived distance range indicates the position of the peak top. The distance range from ESR distance measurement is defined by the region at half-height of the major peak. The distance information for ESR distance measurement and MD simulation on MurD is based on the previous report[1].

Reference

(1) Saio, T.; Hiramatsu, S.; Asada, M.; Nakagawa, H.; Shimizu, K.; Kumeta, H.; Nakamura, T.; Ishimori, K. Conformational ensemble of a multidomain protein explored by Gd^{3+} electron paramagnetic resonance. *Biophys. J.* **2021**, *120* (15), 2943–2951.
